# Epidemiology of intussusception among infants in Ethiopia, 2013-2016

**DOI:** 10.11604/pamj.supp.2021.39.1.21299

**Published:** 2021-07-27

**Authors:** Amezene Tadesse, Fasil Teshager, Goitom Weldegebriel, Ayesheshem Ademe, Eshetu Wassie, Abay Gosaye, Kimberly Pringle, Jason M Mwenda, Umesh D Parashar, Jacqueline E Tate

**Affiliations:** 1 Department of Surgery, Addis Ababa University, Addis Ababa, Ethiopia,; 2 World Health Organization, Ethiopia Country Office, Addis Ababa, Ethiopia,; 3 World Health Organization, Intercountry Support Team, Harare, Zimbabwe,; 4 CDC Foundation, Atlanta, GA, USA,; 5 World Health Organization Regional Office for Africa, Brazzaville, Republic of Congo,; 6 Centers for Disease Control and Prevention, Atlanta, GA, USA

**Keywords:** Intussusception, infants, Ethiopia

## Abstract

**Introduction:**

intussusception is a condition in which one segment of the bowel prolapses into another causing obstruction. Information on the epidemiology of intussusception in sub-Saharan Africa is limited. We describe the sociodemographic and clinical characteristics of children with intussusception in Ethiopia.

**Methods:**

active surveillance for children < 12 months of age with intussusception was conducted at six sentinel hospitals in Ethiopia. Limited socio-economic and clinical data were collected from enrolled children. Characteristics among children who died and children who survived were compared using the Wilcoxon rank sum test for continuous variables and Chi-square tests for categorical variables.

**Results:**

total of 164 children < 12 months of age with intussusception were enrolled; 62% were male. The median age at symptom onset was 6 months with only 12 (7%) of cases occurring in the first 3 months of life. Intussusception was reduced by surgery in 90% of cases and 10% were reduced by enema; 13% of cases died. Compared to survivors, children who died had a significantly longer time to presentation to the first health care facility and to the treating health care facility (median 3 days versus 2 days, p = 0.02, respectively).

**Conclusion:**

the high mortality rate, late presentation of intussusception cases, and lack of modalities for non-surgical management at some facilities highlight the need for better management of intussusception cases in Ethiopia.

## Introduction

A previously licensed rotavirus vaccine in the United States (RotaShield, Wyeth) was associated with an increased risk of intussusception, a condition in which one segment of the intestine prolapses in to another causing obstruction. The risk was estimated at 1 excess intussusception case per 10,000 vaccinated infants, and RotaShield was subsequently removed from the US market [1]. Consequently, the World Health Organization mandated careful monitoring for intussusception with subsequent rotavirus vaccines [2]. Post-licensure evaluations for the two currently widely available rotavirus vaccines, RV1 (Rotarix, GlaxoSmithKline) and RV5 (RotaTeq, Merck), have identified a low-level increased risk of intussusception following rotavirus vaccination in several high and middle income countries [3-7]. However, no increased risk of intussusception was identified in the 7 days following either dose of RV1 in a pooled analysis of data from seven sub-Saharan African countries, including Ethiopia, in the African Intussusception Surveillance Network [8].

Despite no increased risk of intussusception following rotavirus vaccination in Sub-Saharan Africa, information on the epidemiology of intussusception in Ethiopia is limited. A retrospective study to assess patterns and seasonal variation of pediatric intussusception was conducted in Tikur Anbessa Specialized Hospital (TASH) in Addis Ababa from 2011 to 2014 and found that there was a delay in presentation of intussusception cases and the management was almost purely surgical [9]. The study identified 136 cases of intussusception and of which, 77.7% were =2 years-old and the majority (70.8%) were reduced with surgical intervention [9]. Another study done in 2014 at TASH found a substantial increase in non-operative management of intussusception in the hospital. The use of hydrostatic reduction decreased the surgical intervention rate by three fourth and had a success rate of 87% [10]. In this report, we describe the epidemiology of intussusception cases enrolled as part of the African Intussusception Surveillance Network in Ethiopia.

## Methods

**Figure 1:**
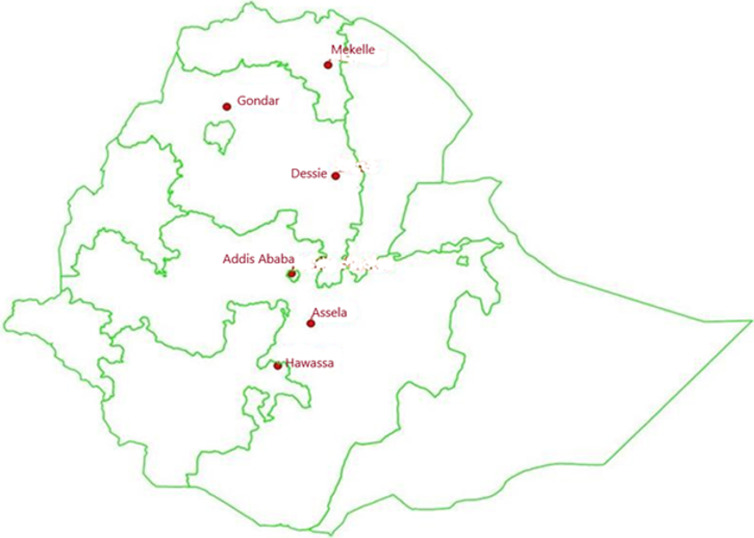
map of Ethiopia and the location of the six surveillance hospitals

Active surveillance for intussusception was conducted at six sentinel tertiary care hospitals across Ethiopia - Assela Teaching Referral Hospital (Assela), Ayeder Referral Teaching Hospital (Mekelle), TASH (Addis Ababa), Dessie Referral Hospital (Dessie), Gondar University Hospital (Gondar), and Hawassa Referral Hospital (Hawassa) - from December 2013 to December 2016, as previously described (Figure 1) [8].

Briefly, the outpatient registry book, the operation logbook, the inpatient registry book were reviewed to identify potential cases of intussusception. Children < 12 months of age with intussusception meeting the Brighton Collaboration criteria for level 1 of diagnostic certainty - intussusception confirmed by radiology, surgery or autopsy -- were enrolled. Staff collected limited socio-economic and clinical data from participants. Date of symptom onset as reported by the parent was taken as date of intussusception onset.

We describe overall sociodemographic characteristics of children with intussusception during the study period as well as characteristics of their clinical course. We also compare these characteristics among children who died and children who survived using the Wilcoxon two sample t-test for continuous variables and Chi-square tests for categorical variables.

**Needs acknowledgment of Gavi funding:** supported by the Gavi Alliance through the CDC Foundation.

## Results

### Descriptive epidemiology of all intussusception patients

**Figure 2:**
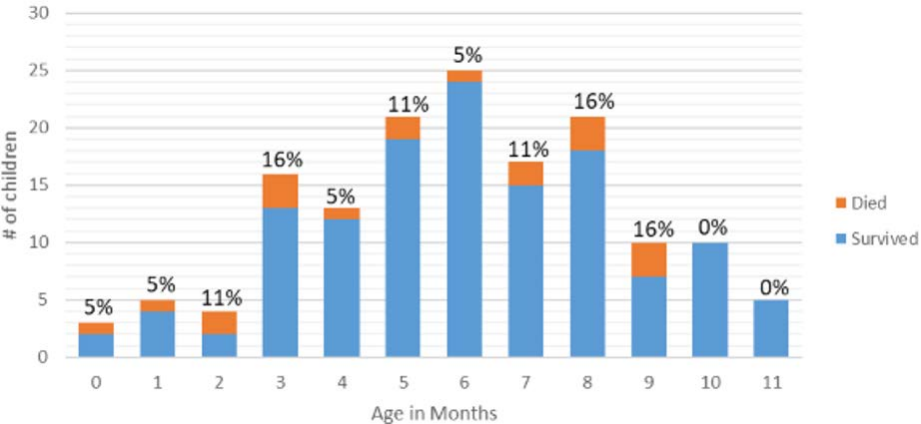
number of children per age in months stratified by outcome (#percentage of the total number of children who died by age)

A total of 164 intussusception cases in children < 12 months of age were enrolled, of which 62% (n = 101) were male. The average age at symptom onset was 6 months (median = 6 months IQR = 4 - 8 months), and 41% of those enrolled were between the ages of 6-8 months (Figure 2). Only 12 (7%) of all intussusception cases during infancy occurred in the first 3 months of life. Over half of participants had electricity, television, or a telephone in their homes (Table 1). Fifty-six percent (n = 74/132) of participants were transferred from the initial healthcare facility where they presented at to a sentinel site, with a median transfer time of 0 days (IQR = 0 - 1). Infants with intussusception had a median of 2 days (IQR = 1-3) of symptoms before presenting to a healthcare facility (Table 1). Intussusception was reduced by surgery (90%; n = 137/153) in the majority of cases with 10% (n = 16) being reduced by liquid or air contrast enema (Table 2). Four of the six sentinel hospitals reduced at least one case by enema, but only one hospital performed more than 5 enema reductions during the study period. Of children with a final disposition (n=151) recorded, 87% (n = 131) were discharged home, 1% (n = 1) were transferred to another facility and 13% (n = 19) died.

**Table 1:** demographic and clinical characteristics of participants stratified by outcome

Characteristic	Total^			Survived			Died			
	N	n	%	N	n	%	N	n	%	p-value*
Male	164	101	62	131	78	60	19	12	63	0.76
Age at Symptom Onset	**N**	**Median**	**IQR**	**N**	**Median**	**IQR**	**N**	**Median**	**IQR**	**p-value**
Age in Months	164	6	4-8	131	6	4-8	19	5	3-8	0.25
# Rooms	140	2	1-3	115	2	1-3	12	2	1-2	0.24
# People	141	5	3-6	115	5	3-6	13	4	4-5	0.84
	N	n	%	N	n	%	N	n	%	p-value
Electricity	127	79	62	104	68	65	11	4	36	0.06
Refrigerator	143	31	22	118	26	22	12	1	8	0.27
Motorcycle	141	3	2	116	2	2	12	0	0	0.65
Radio	145	70	48	119	60	50	13	3	23	0.06
Television	144	72	50	118	61	52	13	6	46	0.71
Telephone	145	77	53	118	64	54	14	8	57	0.84
	**N**	**Median**	**IQR**	**N**	**Median**	**IQR**	**N**	**Median**	**IQR**	**p-value**
Length of symptoms before 1st healthcare facility admission	162	2	1-3	129	2	0-3	19	3	2-5	0.02
Length of symptoms before surveillance hospital admission	162	2	1-4	129	2	1-4	19	3	2-5	0.02
Length of Transfer time between first facility and surveillance hospital	70	0	0-1	58	0	0-1	5	0	0-1	0.76

*P value compared survived versus died **^** All enrolled children including those with unknown disposition

**Table 2:** diagnostic and treatment stratified by outcome

Characteristic	Total			Survived			Died			
	N	n	%	N	n	%	N	n	%	p-value*
Clinical Diagnosis	164	153	93	131	121	92	19	18	95	0.71
Enema	164	19	12	131	16	12	19	3	16	0.66
Ultrasound	164	135	82	131	109	83	19	16	84	0.91
Surgery	164	134	82	131	106	81	19	17	89	0.37
Treatment										
Surgery	153	137	90	124	109	88	16	16	100	0.14
Enema	153	16	10	124	15	12	16	0	0	
Resection†	124	76	61	102	55	54	14	13	93	0.005

* p-value compares survived versus died ^All enrolled children including those with unknown disposition † Among children who had surgery

### Comparison of characteristics of patients who died versus those who survived

Compared with patients who survived, those who died had a similar sex distribution (63% vs 60% male, p = 0.76) and age at onset (median = 5 months vs 6 months; p = 0.25) (Table 1). The sociodemographic characteristics of the two groups were also similar. Notably, compared to survivors, those who died had a significantly longer time of presentation to the first health care facility and to the treating health care facility (median 3 days versus 2 days, p = 0.02, respectively). There were no significant differences in the diagnostic modality used between participants who survived and died (Table 2). No patient who received an enema to reduce the intussusception (n = 16) died. One hospital accounted for 75% of all enemas performed during the study period, and accounted for only 5% of total deaths. Among patients who received surgery as treatment, 12% (n = 15/124) of them died. However, the mortality between enema-reduced and surgically-reduced patients did not reach statistical significance (p = 0.14). Among those who had surgery, children who died had a higher rate intestinal resection (n = 13; 93%) compared with children who survived (n = 55; 54%) (p = 0.005) (Table 2). The three hospitals with the largest mean days of symptoms accounted for 60% (n = 41/68) of intestinal resections during surgery.

## Discussion

Intussusception cases were rare in the first three months of life in Ethiopia with only 7% of all infant cases occurring during this period. The age distribution for intussusception cases in Ethiopia with few cases early in infancy and the number of cases peaking at approximately 6 months of age is similar to that seen in other countries [11]. Rotavirus vaccine is recommended for administration at 6 and 10 weeks of age in Ethiopia and on-time vaccination will ensure that the vaccine is given at a time when the natural risk of intussusception is low.

Significant delays in presentation to health care facilities following onset of symptoms was observed and these delays were associated with an increased risk for mortality. Furthermore, among patients requiring surgery, those who died were more likely to require intestinal resection than those who survived. This is likely because patients who required resection had bowel necrosis and other complications associated with late presentation for treatment. Similar findings have been observed in other African countries [12-15]. Many barriers can contribute to delayed presentations such as rural location, lack of transportation, poverty, patient age, and lack of healthcare literacy. The longer patients delay treatment, the more likely they are to have ischemic bowel, and require surgical resection, which puts patients at risk for mortality.

The mainstay treatment for intussusception in Ethiopia is surgery and 90% of cases in this analysis were treated surgically. While the high surgical rate may reflect clinical need in part due to the late presentation of cases, it may also reflect a reduced comfort with, or lack of availability of, non-surgical management at some facilities. Pediatric surgeons with specialized training and radiographic facilities to guide non-operative management are not available at all hospitals that treat intussusception patients in African countries. Indeed, in our study, one of the six study hospitals accounted for nearly three-quarters of all enema reductions preformed. This aligns with standard of care in the region, including a previous retrospective study done in Ethiopia [9]. Retrospective studies of intussusception in Tanzania and Nigeria showed that 100% and 91% of cases, respectively, were treated surgically with 46% and 9%, respectively, requiring bowel resection [12, 14].

In contrast, enema is often used for diagnosis and treatment of intussusception in the US with success of 83% for pneumatic and 70% for hydrostatic reduction [16]. A 2014 study done at one of our study hospitals, TASH, found that hydrostatic reduction had a success rate of 87% resulting in a decrease in surgical intervention by 75% [10]. Increased availability of radiologic diagnosis and less invasive treatment procedures such as air or hydrostatic reduction enema has resulted in lower mortality rates due to intussusception in many regions of the world [11]. However, high mortality rates have persisted in sub-Saharan Africa, including in Ethiopia, due to lack of qualified personnel for radiologic diagnosis and non-surgical reduction of intussusception [17]. Efforts to increase training in, and availability of facilities for, non-surgical management of intussusception should be explored.

Our analysis has several limitations. First, surveillance was conducted at sentinel teaching and referral hospitals throughout Ethiopia which may not be representative of the diagnosis and treatment of intussusception cases at all hospitals in the country. Similarly, the treatment seeking behaviors of intussusception cases at these facilities may not be representative. Cases from rural areas may die before reaching the hospital and therefore the mortality rate may be underestimated. Finally, the small number of intussusception deaths enabled us to look at crude risk factors for mortality but we were not able to conduct multivariate analyses.

## Conclusion

Rotavirus vaccines were not associated with an increased risk of intussusception in a previous evaluation in sub-Saharan Africa. This analysis found that intussusception cases rarely occur in the first three months of life when rotavirus vaccine is recommended for infants. However, the high mortality rate, late presentation of intussusception cases, and lack of modalities for non-surgical management at some facilities highlight the need for better management of intussusception cases in Ethiopia.

**Funding**: supported by the Gavi Alliance through the CDC Foundation.

### What is known about this topic

- Rotavirus vaccines have been associated with an increased risk of intussusception in some high and middle income countries but not in countries in sub-Saharan Africa;
- Data on the epidemiology of intussusception in sub-Saharan African are sparse.

### What this study adds

- Intussusception rarely occurs in the first three months of life in Ethiopia when rotavirus vaccine doses are given;
- Children with intussusception who die are more likely to present later for treatment than children who survive.
